# Expectations of barriers to psychosocial care: views of parents and adolescents in the community

**DOI:** 10.1007/s00787-015-0717-1

**Published:** 2015-05-13

**Authors:** Marieke Nanninga, Sijmen A. Reijneveld, Erik J. Knorth, Danielle E. M. C. Jansen

**Affiliations:** Department of Health Sciences, University Medical Center Groningen, University of Groningen, Antonius Deusinglaan 1/FA10, 9713 AV Groningen, The Netherlands; Department of Sociology and Interuniversity Center for Social Science Theory and Methodology (ICS), University of Groningen, Groningen, The Netherlands; Department of Special Needs Education and Youth Care, University of Groningen, Groningen, The Netherlands

**Keywords:** Child, Adolescent, Psychosocial care, Barriers to care, Health services accessibility

## Abstract

Parents with a child suffering from psychosocial problems frequently experience barriers to psychosocial care, which may hinder access. Expectations of barriers may have the same effect, but evidence is lacking. The aim of this study is to examine parents’ and adolescents’ expectations of barriers regarding psychosocial care for the child, along with associated child and family characteristics. We obtained data on an age-stratified random sample of school children/pupils aged 4–18 via questionnaires (*N* = 666; response rate 70.3 %). Expectations of barriers to psychosocial care were measured with the “Barriers to Treatment Participation Scale-Expectancies” questionnaire (BTPS-exp). Results showed that 64 % of the parents of children below age 12, 59 % of the parents of adolescents (age 12–18), and 84 % of the adolescents expected one or more barriers. Parents and adolescents expected barriers most frequently with respect to irrelevance of treatment. Mainly parents with low educational level and their adolescents expected barriers regarding treatment, and quite a few characteristics of parents of adolescents were associated with expecting multiple barriers regarding treatment demands and issues, for example, single parents, parents of lower educational level and of adolescent boys, and parents of adolescents with psychosocial problems. We conclude that adolescents especially, but also their parents and parents of younger children, expect major barriers to psychosocial care, which may greatly hinder appropriate care seeking. This evidence may support professionals and policymakers in their attempts to improve access to psychosocial care.

## Introduction

Only a minority of the children and adolescents with emotional or behavioral problems receive psychosocial care [1–12]. For example, a recent study showed that, of the adolescents in need of psychosocial care, only 29 % actually received that care [11]. One of the causes may be the barriers to care that parents and children expect or have experienced [13, 14]. According to Owens and colleagues, barriers to care comprise those “factors that have prevented access or created difficulties in accessing child mental health services” (p.731) [15]. Between 35 and 61 % of parents with a child suffering from psychosocial problems experience barriers to care [15, 16]. These barriers can be structural—financial, for example—but can also relate to perceptions of care or psychosocial problems: the prospect of encountering inadequate care providers or the expectation that problems will improve by themselves, for example [15, 17].

Most evidence about barriers to care is based on clinical samples, i.e., children and adolescents with psychosocial problems either in need of or using psychosocial care [15–21]. This evidence shows that the barriers experienced by those in need have been shown to be associated with a lower intention of seeking help [18], along with a lower likelihood of using psychosocial care [19–21]. Barriers experienced by parents during their child’s treatment have been shown to be associated with poor care outcomes: higher dropout rates, less symptom improvement, and less treatment acceptance by the child and parent [17, 22, 23].

To our knowledge, community-based evidence is lacking in terms of expectations that children, adolescents, and parents have about barriers to children’s psychosocial care. These expectations are likely to influence care seeking and use. For example, according to Andersen’s *Health Behaviour Model*, expectations regarding health care are among the determinants for patients’ utilization of care [24–26]. Furthermore, the review of Morrisey-Kane and Prinz concluded that positive parental expectations towards care are of importance for successful help seeking and engagement in treatment [14].

The aim of our study was to examine in a community sample of children and adolescents: (1) the number of barriers that parents and adolescents expect when considering seeking psychosocial care for their child or for themselves, respectively; (2) the type of barriers expected most frequently; and (3) the child and family characteristics associated with these expectations. In this study, psychosocial care is defined as all care aimed at reducing or making manageable psychosocial problems of children and adolescents [27]. In the Netherlands, like in many other countries, this is provided by preventive child health care, child and adolescent social care, and mental health care [28–30]. Knowledge about the expectations of parents and adolescents regarding barriers to care may provide direction to improve the help-seeking and treatment process.

## Methods

### Study design

We used data from the first measurement wave of the community sample of a large prospective cohort study called TakeCare [28, 31]. TakeCare is conducted by the Collaborative Centre on Care for Children and Youth (C4Youth) and is designed to investigate the trajectories and outcomes of children aged 4–18 receiving psychosocial care in one Dutch region. The design was assessed by the Medical Ethical Committee of the University Medical Center Groningen, and approved without needing full assessment. Informed consent was obtained from all participating respondents.

### Sample and procedure

We used a stratified random sample of school children and pupils (*N* = 1025), obtained via five primary schools, two secondary schools, and one school for intermediate vocational education, recruited by taking into account the distribution of children and adolescents across the study region according to their age, gender, socioeconomic position, and degree of urbanization. Parents/caregivers of children aged 4–18 years old and adolescents (age ≥12) were invited to participate between April 2011 and June 2013. Children with insufficient understanding of Dutch, living outside the northern region, or following special education because of intellectual disability were excluded (*N* = 77). Of the eligible 948 respondents, 666 participated, i.e., either the child and/or the parent (response 70.3 %). The main reasons for non-participation were opting out (*N* = 99). Differences between respondents and non-respondents were small for age, gender, degree of urbanization, and severity of psychosocial problems (the latter based on one impact-question of the Strengths and Difficulties Questionnaire (SDQ) [32]), with Cohen’s effect sizes ranging from 0.02 (psychosocial problems) to 0.08 (degree of urbanization). Differences between respondents and children in the community were small for age and gender, with effect sizes being 0.00 and 0.01, respectively [28].

Data were obtained from parents/caregivers and adolescents via web-based or paper questionnaires, and, if needed, we provided assistance in filling out the questionnaire. Participants were frequently reminded about filling out the questionnaire, and returned questionnaires were checked for completeness to reduce the chance of missing data. Participants were rewarded with a gift card worth ten euros.

### Measures

Parents’ and adolescents’ expectations of barriers to care were measured using the Barriers to Treatment Participation Scale-Expectancies (BTPS-exp) [17, 33], translated into Dutch following the Guillemin translation procedure [34]. Parents and adolescents were asked to “imagine that you are seeking psychological help, counseling, or advice [for your child]” and asked to indicate to what extent they agreed with the items, with answer categories ranging from “totally disagree” to “totally agree” (5-point Likert scale; 44 items in the parent version, 43 items in the adolescent version). An expected barrier was coded as occurring when rated with “somewhat agree” or “totally agree” to calculate the total number of expected barriers. In addition, we calculated mean scores for the total scale and for each of the subscales:

- Stressors and obstacles competing with treatment, i.e., problems regarding transport, other children at home, activities, health, or conflict with a significant other about coming to treatment (parent and adolescent versions 20 and 19 items, respectively)
- Treatment demands and issues, i.e., concerns about treatment cost and duration, having a voice in treatment, confusing information (10 items)
- Perceived irrelevance of treatment, i.e., concerns about the need for and relevance of treatment, about treatment introducing new or other problems (8 items)
- Problematic relationship with therapist, i.e., concerns about not having a good relationship with the therapist, not receiving enough support (6 items).

Internal consistencies of the total scale and subscales of the parent and adolescent versions were good (lowest Cronbach α = 0.83).

### Child characteristics

Relevant child characteristics included age, gender, ethnicity, psychosocial problems, and past psychosocial care use. Ethnicity was defined as either Dutch or non-Dutch (i.e., the child and/or one of the parents was foreign-born). Children’s psychosocial problems were measured using the total difficulties score of the “Strengths and Difficulties Questionnaire” (SDQ-TDS), based on the past 6 months (Cronbach’s α parent version = 0.80; adolescent version = 0.72) [31, 35–37]. The score consists of 20 items describing positive and negative attributes of children on the following dimensions: emotional symptoms, conduct problems, hyperactivity/inattention, and peer problems. The scale was dichotomized into the “normal” and the “borderline to abnormal” range of the SDQ.

Psychosocial care use in past 6 months was measured using the “Questionnaire Intensive Care for Youth” [38–40]. We asked parents and adolescents to indicate among a list of professionals and types of care whether they had contact because of psychosocial problems. A child using psychosocial care was defined as one whose parent (or the adolescent him/herself) indicated that they or the child had contacted a professional (i.e., a general practitioner, psychologist, or psychiatrist) and/or used care (i.e., outpatient social care, day or residential treatment, foster care) for psychosocial problems of the child in the past 6 months.

### Family characteristics

The family characteristics included were parental educational level and family composition. Parental educational level was based on the highest educational level achieved by either one of the parents/caregivers [41]. The categories “primary education” and “lower levels of secondary education” were combined into one category, because only a few parents fell into the first category.

Family composition was assessed by asking the parent with whom the child lived. Answers were categorized as “two-parent family” or “other” (e.g., living with one parent, a foster family, or living in a residential care facility).

### Statistical analyses

We first described the background characteristics of the sample, for parents of children (aged 4–12), parents of adolescents (aged 12–18), and adolescents separately. Next, we assessed their number of expectations regarding barriers to care (aim 1), and the types of barriers most frequently expected (aim 2). Differences between the scores of parents and adolescents were calculated using Pearson Chi-square tests and non-parametric Mann–Whitney *U* tests because of the scales’ skewed distributions.

We performed univariable logistic regression analyses to assess the crude associations of child and family characteristics with expecting multiple barriers of each type. In addition, we performed multivariable logistic regression analyses to assess the adjusted associations (aim 3). Both crude and adjusted analyses were performed with Generalized Estimating Equations modeling (GEE) (with exchangeable correlation structure) to account for possible inter-correlations between children from the same school. The BTPS-exp subscales were dichotomized as the 25 % highest scores vs. lower scores (Table 1) because of the skewed distributions. For all logistic regression analyses, odds ratios (OR) and 95 % confidence intervals (CI) were presented. A *p* value <0.05 was considered statistically significant (two-sided test). Analyses were performed using SPSS Statistics version 20.

**Table 1:** Range of the 25 % highest scores for the subscales of the BTPS-exp

Score (possible scores range from 1 to 5)	Parents children <12 years		Parents adolescents		Adolescents	
	From	To	From	To	From	To
Stressors and obstacles	1.55	5.00	1.45	5.00	2.53	5.00
Demands and issues	2.44	5.00	2.50	5.00	2.78	5.00
Perceived irrelevance	3.00	5.00	3.00	5.00	3.29	5.00
Problematic relationship with therapist	2.60	5.00	3.00	5.00	3.00	5.00

## Results

### Sample characteristics and barrier expectations

Based on parent reports, 10.9 % of the children and 10.8 % of the adolescents had psychosocial problems, and 26.9 % and 30.2 % of all children and adolescents had used psychosocial care in the past 6 months, respectively. Of the adolescents, 21.1 % reported psychosocial problems, and 34.6 % reported use of psychosocial care in the past 6 months (Table 2). The majority of the parents expected one or more barriers, i.e., 63.6 and 58.9 % of the parents of children and adolescents, respectively. Of the adolescents, 83.9 % expected one or more barriers (Table 3).

**Table 2:** Child and family characteristics of the participants

Characteristics	Parents children <12 years		Parents adolescents		Adolescents	
	*N* = 368^a^		*N* = 278^b^		*N* = 280^b^	
	*N*	(%)	*N*	(%)	*N*	(%)
Child characteristics						
Gender						
Male	163	44.3	127	45.7	116	41.4
Female	205	55.7	151	54.3	164	58.6
Ethnicity						
Dutch	336	93.6	242	89.3	233	90.3
Non-Dutch	23	6.4	29	10.7	25	9.7
Psychosocial problems						
Normal	328	89.1	248	89.2	221	78.9
Borderline	15	4.1	12	4.3	33	11.8
Abnormal	25	6.8	18	6.5	26	9.3
Psychosocial care use in past 6 months						
No	269	73.1	194	69.8	183	65.4
Yes	99	26.9	84	30.2	97	34.6
Family characteristics						
Parental educational level						
Primary education	2	0.5	1	0.4	1	0.4
Lower levels of secondary education	21	5.7	31	11.2	25	9.7
Higher levels of secondary education	189	51.5	126	45.7	119	46.1
Senior vocational education	119	32.4	83	30.1	78	30.2
University	36	9.8	35	12.7	35	13.6
Family composition						
Biological two-parent family	252	68.7	166	59.7	212	75.7
Other	115	31.3	112	40.3	68	24.3

^a^Numbers do not always add up to *N* = 368 due to missing data

^b^ *N* = 258 (86.0 %) couples of parents and adolescents participated, *N* = 20 (6.7 %) parents participated without participation of the adolescent, and *N* = 22 (7.3 %) adolescents participated without participation of the parent

**Table 3:** Parents’ and adolescents’ barrier expectations: frequencies and means

Expectations of barriers	Parents children <12 years		Parents adolescents		Adolescents	
	*N*	(%)	*N*	(%)	*N*	(%)
Number (range 1–44^a^)						
0	133	(36.3)	114	(41.0)	45	(16.2)^c,^**
1	88	(24.0)	61	(21.9)	31	(11.2)^c,^**
2	44	(12.0)	29	(10.4)	25	(9.0)^c,^**
≥3	101	(27.6)	74	(26.6)	177	(63.7)^c,^**
Total number (M, SD)	1.99	(2.9)	2.32	(3.7)	6.28	(6.6)^c,^**
Score (range 1–5)	M	(SD)	M	(SD)	M	(SD)
Barriers total	1.63	(0.5)	1.67	(0.6)	2.11	(0.7)^c,^**
Stressors and obstacles	1.35	(0.5)	1.33	(0.5)^b,#^	1.91	(0.8)^c,^**
Demands and issues	1.72	(0.8)	1.76	(0.8)	2.05	(0.8)^c,^**
Perceived irrelevance	2.12	(0.8)	2.23	(0.9)	2.62	(0.9)^c,^**
Problematic relationship with therapist	1.76	(0.8)	1.95	(0.9)^b,^*	2.09	(0.9)^c,#^

*M* mean, *SD* standard deviation

^a^Range 1–43 for adolescents

^b^Significant differences between parents of children <12 years and parents of adolescents, ^# ^<0.10; * *p* < 0.05

^c^Significant differences between parents of adolescents and adolescents, ^# ^<0.10, * p < 0.05, ** p < 0.001

Regarding the type of barrier expectations, both parents and adolescents expected barriers of the type “perceived irrelevance of treatment” most frequently, followed by “problematic relationship with therapist,” “treatment demands and issues,” and, lastly, “stressors and obstacles competing with treatment” (Table 3).

Overall, no significant differences were found between the scores of parents of children and parents of adolescents, except for the subscale “problematic relationship with therapist.” Scores of adolescents were statistically significantly higher than scores of their parents, except for scores on the subscale “problematic relationship with therapist.”

### Association of child and family characteristics with barrier expectations

The results of univariable logistic regression analyses showed that, among the three groups, there were a series of variables associated with expecting different types of multiple barriers. This was particularly true of “stressors and obstacles competing with treatment”, “treatment demands and issues” and “problematic relationship with therapist” (Table 4).

**Table 4:** Association of child and family characteristics with expecting different types of multiple barriers: results of logistic regression analyses with generalized estimating equations modeling (GEE) for parents of children <12, parents of adolescents, and adolescents, separately

Characteristics	Stressors and obstacles		Demands and issues		Perceived irrelevance		Problematic relationship with therapist	
	Crude	Adjusted	Crude	Adjusted	Crude	Adjusted	Crude	Adjusted
	OR (CI)	OR (CI)	OR (CI)	OR (CI)	OR (CI)	OR (CI)	OR (CI)	OR (CI)
**Parents of children <12 years**								
Child’s gender (female vs. male)	0.77 (0.43–1.37)	0.80 (0.44–1.43)	1.22 (0.64–3.33)	1.17 (0.59–2.30)	0.82 (0.65–1.02)^#^	0.79 (0.57–1.10)	**1.45 (1.14**–1.85)**	1.36 (0.95–1.94)^#^
Child’s ethnicity (non-Dutch vs. Dutch)	1.18 (0.71–1.95)	1.12 (0.65–1.91)	0.67 (0.21–2.10)	0.76 (0.24–2.40)	0.83 (0.49–1.43)	0.86 (0.42–1.71)	0.46 (0.13–1.57)	0.56 (0.13–2.47)
Child’s psychosocial problems (borderline/abnormal vs. normal)	**1.70 (1.10**–2.62)*	1.27 (0.76–2.15)	1.68 (0.82–3.41)	1.94 (0.65–5.78)	0.66 (0.24–1.80)	0.62 (0.29–1.34)	0.94 (0.52–1.72)	0.92 (0.52–1.61)
Psychosocial care use in past 6 months (use vs. no use)	**1.85 (1.06**–3.23)*	1.78 (0.82–3.85)	1.67 (0.77–3.64)	0.94 (0.50–1.75)	0.84 (0.36–1.96)	0.89 (0.39–2.05)	0.86 (0.60–1.23)	0.97 (0.65–1.44)
Parental educational level (reference: university)								
Primary/Secondary, lower levels	**2.35 (1.22**–4.53)*	**2.25 (1.50**–3.36)***	**6.41 (2.49**–16.55)***	**5.35 (1.68**–17.08)**	1.93 (0.91–4.08)^#^	1.74 (0.75–4.03)	**2.96 (1.16**–7.57)*	2.74 (0.79–9.45)^#^
Secondary, higher levels	1.82 (0.72–4.57)	**2.21 (1.09**–4.49)*	**3.70 (1.41**–9.69)**	**3.32 (1.08**–10.21)*	1.14 (0.54–2.39)	1.22 (0.47–3.19)	**3.03 (1.14**–8.08)*	2.73 (0.79–9.45)
Senior vocational	1.23 (0.63–2.41)	1.47 (0.95–2.27)^#^	**3.39 (1.16**–9.92)*	2.88 (0.81–10.27)^#^	1.04 (0.47–2.30)	1.06 (0.43–2.62)	2.23 (0.84–5.94)	1.98 (0.62–6.33)
Family composition (other vs. biological two-parent family)	1.10 (0.83–1.45)	0.89 (0.51–1.56)	0.68 (0.28–1.67)	0.64 (0.29–1.41)	0.96 (0.68–1.35)	0.98 (0.73–1.31)	0.91 (0.68–1.21)	0.92 (0.66–1.28)
**Parents of adolescents**								
Child’s gender (female vs. male)	**0.51 (0.45**–0.57)***	**0.41 (0.29**–0.57)***	**0.44 (0.35**–0.56)***	**0.42 (0.35**–0.50)***	0.63 (0.33–1.20)	0.61 (0.32–1.14)	0.73 (0.53–1.01)^#^	**0.67 (0.52**–0.86)**
Child’s ethnicity (non-Dutch vs. Dutch)	**4.58 (2.41**–8.72)***	**4.88 (2.90**–8.21)***	1.45 (0.96–2.18)^#^	1.33 (0.79–2.24)	**2.18 (1.48**–3.22)***	**2.27 (1.43**–3.59)**	**1.62 (1.35**–1.94)***	**1.53 (1.16**–2.00)**
Child’s psychosocial problems (borderline/abnormal vs. normal)	2.11 (0.83–5.34)	1.34 (0.90–2.00)	**3.45 (1.39**–8.58)**	**2.77 (1.59**–4.82)***	0.65 (0.36–1.18)	0.65 (0.42–1.01)^#^	0.91 (0.64–1.28)	0.94 (0.42–2.08)
Psychosocial care use in past six months (use vs. no use)	1.72 (0.92–3.21)^#^	1.39 (0.85–2.26)	1.31 (0.53–3.24)	0.84 (0.32–2.23)	0.93 (0.42–2.09)	0.92 (0.43–1.94)	0.76 (0.51–1.14)	0.58 (0.34–1.01)^#^
Parental educational level (reference: university)								
Primary/Secondary, lower levels	0.87 (0.31–2.40)	0.72 (0.30–1.72)	**2.59 (1.52**–4.41)***	2.48 (0.79–7.77)	**2.10 (01.29**–3.43)**	**2.14 (1.60**–2.86)***	**4.85 (2.21**–**10.67**)***	**4.79 (1.65**–13.91)**
Secondary, higher levels	1.55 (0.61–3.95)	1.50 (0.61–3.68)	1.82 (0.94–3.54)^#^	**1.92 (1.32**–2.80)**	**2.49 (1.66**–3.72)***	**2.66 (1.72**–4.11)***	**3.01 (1.85**–4.90)***	**2.94 (1.84**–4.68)***
Senior vocational	1.15 (0.65–2.02)	1.13 (0.74–1.73)	1.33 (0.83–2.12)	1.36 (0.77–2.40)	1.40 (0.88–2.23)	1.29 (0.91–1.85)	**2.21 (1.23**–3.99)**	**1.98 (1.12**–3.50)*
Family composition (other vs. biological two-parent family)	1.77 (0.97–3.23)^#^	**1.70 (1.15**–2.50)**	**1.88 (1.06**–3.34)*	**1.86 (1.21**–2.85)**	0.87 (0.65–1.18)	0.93 (0.74–1.18)	1.27 (0.87–1.85)	1.33 (0.92–1.90)
**Adolescents**								
Child’s gender (female vs. male)	**1.59 (1.23**–2.05)***	**1.37 (1.03**–1.82)*	0.80 (0.61–1.05)	**0.71 (0.54**–0.93)*	0.70 (0.42–1.16)	0.69 (0.43–1.12)	1.21 (0.99–1.47)^#^	1.09 (0.78–1.51)
Child’s ethnicity (non-Dutch vs. Dutch)	1.16 (0.60–2.23)	0.84 (0.40–1.78)	0.75 (0.40–1.40)	0.61 (0.25–1.50)	1.35 (0.65–2.80)	1.30 (0.99–1.71)^#^	0.80 (0.36–1.79)	0.83 (0.34–2.07)
Child’s psychosocial problems (borderline/abnormal vs. normal)	**2.26 (1.87**–2.74)***	**2.12 (1.67**–2.70)***	1.28 (0.98–1.67)^#^	1.15 (0.79–1.68)	**1.83 (1.42**–3.37)***	1.54 (0.92–2.58)	**2.73 (1.91**–3.88)***	**3.83 (1.97**–7.43)***
Psychosocial care use in past 6 months (use vs. no use)	**2.00 (1.64**–2.44)***	**1.57 (1.13**–2.20)**	**1.47 (1.17**–1.85)**	**1.60 (1.23**–2.07)***	1.21 (0.88–1.68)	1.03 (0.76–1.38)	0.92 (0.62–1.37)	0.57 (0.29–1.11)^#^
Parental educational level (reference: university)								
Primary/Secondary, lower levels	0.50 (0.14–1.76)	0.49 (0.09–2.70)	1.38 (0.76–2.50)	1.15 (0.57–2.31)	**2.82 (1.51**–5.30)*	**2.58 (1.04**–6.42)*	1.84 (0.68–5.00)	**2.36 (1.49**–3.72)***
Secondary, higher levels	**0.50 (0.33**–0.76)*	0.53 (0.23–1.03)^#^	**1.67 (1.19**–2.34)**	**1.53 (1.16**–2.02)**	1.15 (0.76–1.75)	1.07 (0.77–1.49)	**2.89 (2.18**–3.95)***	**3.39 (2.48**–4.63)***
Senior vocational	**0.34 (0.23**–0.52)***	**0.36 (0.22**–0.58)***	0.51 (0.24–1.10)^#^	0.45 (0.20–1.00)^#^	0.56 (0.31–1.03)^#^	**0.51 (0.26**–0.99)*	1.59 (0.77–3.26)	**1.95 (1.04**–3.63)*
Family composition (other vs. biological two-parent family)	0.86 (0.46–1.63)	0.72 (0.26–2.03)	1.21 (0.52–2.87)	1.08 (0.35–3.29)	1.28 (0.82–2.00)	1.16 (0.73–1.85)	**1.38 (1.09**–1.75)**	1.05 (0.62–1.79)

All significant odds ratios are in bold

*OR* odds ratio, *CI* confidence interval

^#^<0.10; * p < 0.05; ** p < 0.01; *** p < 0.001

The results of multivariable logistic regression analyses showed that expecting multiple barriers of the type “stressors and obstacles” was more likely in parents of children with a low educational level (Table 4). For parents of adolescents, this was more likely when their child was a boy, was of non-Dutch ethnicity or from other than two-parent families. For adolescents themselves, this was more likely for girls, when they had psychosocial problems, had used psychosocial care previously and when their parents had a university educational level (in contrast with a senior vocational education).

Expecting multiple barriers of the type “treatment demands and issues” was more likely for parents of children with a low parental educational level. For parents of adolescents, this was more likely for parents of adolescent boys, of adolescents with psychosocial problems, parents with higher levels of secondary education (compared to university level), and for parents in other than a biological two-parent family. For adolescents, this was more likely for boys, for those who had used psychosocial care previously and for those who had parents with a higher secondary educational level (compared to university level).

Expecting multiple barriers of the type “perceived irrelevance of treatment” was, in the adjusted models, not statistically significantly associated with any of the child and family characteristics for parents of children. For parents of adolescents, this type of expected barriers was more likely when they were of non-Dutch ethnicity or had low education. For adolescents, it was more likely with parents who had primary or lower levels of secondary education (compared to university) and for adolescents with parents who had university education compared to senior vocational education.

Lastly, when it came to expecting multiple barriers of the type “problematic relationship with therapist,” none of the examined characteristics of parents of children were significantly associated. For parents of adolescents, expecting multiple barriers of this type was more likely in parents of boys, of non-Dutch ethnicity and with low and medium educational level; for adolescents this was more likely when they had psychosocial problems and when their parents had low and medium educational level.

## Discussion

Our community-based study showed that the majority of parents and adolescents expected barriers to child and adolescent psychosocial care. Adolescents expected barriers more frequently than their parents did. The most frequently expected barriers were those with respect to treatment irrelevance, problematic relationship with therapists, and treatment demands. Several child and family characteristics were associated with almost all types of barriers, except for irrelevance of treatment. It was mainly parents with low educational level and their adolescents who expected barriers regarding treatment. Especially for parents of adolescents quite a few characteristics were associated with expecting multiple barriers regarding treatment demands and issues, e.g., single parents, with lower educational level, parents of male adolescents, and of adolescents with psychosocial problems.

### Interpretation and fit with other studies

To our knowledge, this study is the first that assessed parents’ and adolescents’ expectations of barriers to child and adolescent psychosocial care. Findings on the number and types of expected barriers point in the same direction as the findings for studies on barriers experienced in accessing care, except for one study that showed a much smaller number of perceived barriers [15, 16]. The latter might have been specific to a mainly African-American low-income sample, or might simply imply that expectations and actual experiences of barriers differ.

Our study showed that the number of children and adolescents who used psychosocial care in the last 6 months was higher than the number that reported having psychosocial problems. This seems to be in contrast with findings of studies so far [3, 11]. It might suggest that psychosocial problems of children and adolescents are reduced due to successful treatment. However, information about causality is lacking because both problems and psychosocial care use refer to the same time period. The finding might also be explained by the fact that our definition of psychosocial care use is rather broad: we measured any care aimed at reducing or making manageable psychosocial problems of children and adolescents. For example, adolescents mainly use general care for psychosocial problems instead of specialized social or mental health care [29]. It is quite likely that (parents of) some children and adolescents consulted general care providers because of worries about psychosocial issues which are not necessarily scored as problems on the SDQ.

Regarding barriers, adolescents expected barriers more frequently than their parents did, a finding which implies that it is worthwhile to assess the expectations of both. This is consistent with evidence showing that parents and their children often differ in their views, including the well-known example of their views on psychosocial problems [42–44]. It might be that children in general foresee barriers more often than their parents do, because they are the ones who have to follow treatment and have to address their problems. This may be especially true for adolescents. Their developmental stage is characterized by a growth in autonomy, in self-directedness, and in the desire to solve problems on their own, which is brought about by creating distance from and less dependency on their parents and educators [45–47]. This may lead to an avoidance of interference from others, such as psychosocial care professionals, which might be reflected in the expectation of multiple barriers to care.

The most frequently expected type of barrier was the expectation that treatment would be irrelevant. For parents of children this result emerged regardless of sociodemographic background. This might reflect that these parents prefer to solve problems on their own or within their informal networks. It might also mean that there is a lack of confidence in the quality of the treatment and its effectiveness [47]. Parents of adolescents of non-Dutch ethnicity more often expected this type of barriers as well as barriers regarding the relationship with the therapist and stressors and obstacles at home. It might also be that parents from some cultures expect or have experienced resistance to arranging psychosocial care for the child more often, because of a greater stigma attached to psychosocial problems and to using psychosocial care [48–50].

Barriers regarding treatment—i.e., irrelevance, problematic relationship with therapists, and demands and issues—were more frequently expected by parents of low educational level and their adolescents. These families, as compared to families with higher educational level, more often lack the skills needed to navigate the mental health care or social care systems, which might explain our finding. These families more often have limited health literacy, for example, which is a factor that has been shown to be associated with distrust of professionals and pessimism about treatment [51].

Furthermore, barriers related to treatment demands and issues were expected by parents and adolescents who already (might) had some experience with seeking psychosocial care. That is by parents of adolescents with psychosocial problems and adolescents who had used psychosocial care in the past. Also, adolescents who had psychosocial problems expected barriers regarding a problematic relationship with a therapist. It might be that seeking or using psychosocial care is actually the current reality for them. Their expectations might well be based on actual experiences, which is something parents and adolescents do not have if they are just imagining having to seek psychosocial care [52]. This explanation might also hold for our other findings, for example on the association with parental educational level, non-Dutch ethnicity and one-parent families because these children also are more at risk and more often show psychosocial problems [12, 48, 53–55].

Barriers of single parents, i.e., regarding treatment demands and stressors at home, might, for example, be based on the burden they experience from having to organize the household on their own, or on a lack of finances to meet treatment costs. Parents of adolescent boys, who expected barriers regarding treatment demands and a problematic relationship with a therapist, might for example, more often be apt to expect that their child would refuse to attend treatment, simply as an expression of the more externalizing behavior pattern that boys show [56], as compared to parents of female adolescents. This might also be the reason why adolescent boys themselves expected these barriers.

Barriers related to stressors and obstacles that compete with treatment, which are mainly dependent on the family’s home situation, were expected on the part of parents of adolescent boys, and of one-parent families, of adolescents with psychosocial problems and/or who had used psychosocial care in the past. In addition, these findings might be explained by previously experienced barriers. However, it remains unclear why adolescent girls mainly expect this type of barriers, whereas the association is the other way around for their parents.

Lastly, adolescents with parents with a university degree were especially likely to expect this type of barrier, which, for instance, might be explained by a lack of time to attend treatment due to more leisure time activities, such as sports and music, compared to children from lower educated parents [57].

### Strengths and limitations

A strength of our study is that it is the first community-based evidence regarding parents’ and adolescents’ expectations of barriers to child and adolescent psychosocial care. Another strength is its extensive recruitment procedure, the successful actions to reduce missing data, and the relatively high response rate.

A limitation of our study might be a potential selection bias. However, we found only small differences between respondents and non-respondents, and between respondents and children in the community, which thus decreases the likelihood of bias. A final limitation is that we did not adjust for correlation of data between adolescents and parents by accounting for the dyadic nature of the data [58, 59]. This is unlikely to have affected our findings in a major way.

### Implications

This study showed that adolescents especially, but also their parents and parents of younger children, expect substantial barriers to psychosocial care, which may greatly hinder appropriate care seeking. Barriers are mainly expected regarding treatment, and some groups of parents and adolescents expect barriers more frequently than others do. This evidence may provide support for professionals and policymakers in their attempts to improve access to psychosocial care. For example, intake professionals in treatment settings might address and try to find solutions for barriers among children and their parents who received previous care.

Professionals and policymakers will also, however, need additional evidence concerning the determinants of expectations of barriers, since our study was only able to explain a part of this variation. Major issues pertinent to this might involve the skills that parents and adolescents needed to navigate care as well as personality characteristics and coping styles. Furthermore, it has yet to be confirmed whether expectations of barriers are indeed predictive of actually experiencing barriers later on in the process of entering psychosocial care. Still, a mitigation of these barrier expectations might greatly increase the access to care of those who need it most. Finally, to support specific types of care, insights should be obtained on barriers related to specific types of care or treatment.
